# Prevalence and pattern of cognitive impairment in rural and urban populations from Northern Portugal

**DOI:** 10.1186/1471-2377-10-42

**Published:** 2010-06-11

**Authors:** Belina Nunes, Ricardo D Silva, Vitor T Cruz, Jose M Roriz, Joana Pais, Maria C Silva

**Affiliations:** 1 Serviço de Neurologia, Hospital Pedro Hispano, 4464-513 Senhora da Hora, Portugal; 2 Centro de Estudos de Demências, 4100-440, Porto, Portugal; 3 Serviço de Neurologia, Hospital de S.Sebastião, 4520-211 Santa Maria da Feira, Portugal; 4 Laboratório de Neuropsicologia, Hospital de S.Sebastião, 4520-211 Santa Maria da Feira, Portugal; 5 UNIFAI, Instituto de Ciências Biomêdicas Abel Salazar (ICBAS), University of Porto, 4099-003, Porto, Portugal

## Abstract

**Background:**

Despite worldwide recognition of the burden of dementia, no epidemiological data is yet available in Portugal. The objective of this study is to estimate the prevalence and describe the pattern of cognitive impairment with dementia or no dementia (CIND) in rural and urban populations from Northern Portugal.

**Methods:**

Two random samples of residents aged 55 to 79 years in rural and urban communities were drawn from the health centres registries to be screened for cognitive impairment. The screening criteria for dementia were an abnormal Mini-Mental State Examination (MMSE) score or a Blessed Dementia Scale score. After excluding those who tested positive for dementia, cut-off points for CIND were set at 1 standard deviation below the mean of the MMSE according to educational level. All those who screened positive either for dementia or CIND were examined by a neurologist for establishing a definitive diagnosis.

**Results:**

The prevalence of cognitive impairment was higher in rural than in urban populations, 16.8% (95% CI: 14.3-19.8%) vs. 12.0% (95%CI: 9.3-15.4%), with a rural/urban prevalence ratio (PR) of 2.16 (95% CI: 1.04-4.50) in the eldest and 2.19 (95% CI: 1.01-4.76) in persons with vascular risk factors. The prevalence of dementia was 2.7% (95% CI: 1.9-3.8%) with a rural/urban PR = 2.1 and the prevalence of CIND was 12.3% (95% CI: 10.4-14.4%) and PR = 1.3. The prevalence of dementia increases exponentially with age and in those with cerebrovascular disease or other comorbid conditions while the prevalence of CIND, besides these factors, is also higher in persons with low levels of education or vascular risk factors. Alzheimer's and vascular disease were equally likely aetiologies of dementia (38.7%), the later more common in men PR(F:M = 0.3) as opposed to the former PR(F:M = 2.0). Vascular CIND, associated either with cerebrovascular disease or vascular risk factors was more frequent (39.7%) then depression (18.4%) or any other aetiology.

**Conclusions:**

The prevalence of cognitive impairment is higher in rural compared with urban populations. This is shown in the synergy between age and rurality, with the rural/urban prevalence ratio increasing with age. In this relatively young population from Northern Portugal, cerebrovascular disease as well as vascular risk factors account for 48% of overall cognitive impairment.

## Background

Dementia, as defined by the American Psychiatric Association's 4th revision of the Diagnostic and Statistic Manual of Mental Disorders (DSM-IV-TR), is characterized by the presence of cognitive deficits over multiple domains, including memory and at least another - aphasia, apraxia, agnosia or executive functioning disturbance - which should represent a deterioration from a previously better level of functioning, exceeding what would be reasonably expectable for normal aging, and be severe enough to compromise the subject's occupational and social skills [1]. Although not essential for diagnosis, several other cognitive domains can be variably affected - as disturbances of visual-spatial processing, insight, critical judgement, attention or social inhibition, perceptual or self neglect, aggressiveness and behavioural changes, humour and sleep disturbances, anxiety, delusions and hallucinations, motor or gait disturbances, etc. - and the diagnosis must be withheld if the cognitive deficits are known to occur exclusively in the dependence of delirium/acute confusional states or a major depressive disorder. Accordingly, not all cognitively impaired people have dementia, and a number of concepts have been proposed to describe the transitional states between cognitive integrity, subjective memory complaints, physiological mental aging and dementia. 'Cognitive impairment no dementia' (CIND) is perhaps the most comprehensive and unifying, by merely including all persons suffering from cognitive disturbances not severe enough to satisfy the diagnostic criteria for dementia [2,3].

Understanding the epidemiology of dementia and CIND in a given population is crucial for an adequate planning of public health strategies and rational allocation of resources. However, given the lack of epidemiological data on dementia in Portugal, the number of affected persons has been calculated using prevalence estimates in other European countries [4,5], which may not reflect the patterns of disease in our country. For instance, stroke and TIA incidences in rural northern Portugal are known to rank among the highest reported in community-based studies [6,7], probably resulting from the high prevalence of arterial hypertension [8], with a higher risk for cognitive impairment, either of Alzheimer or vascular type, associated with vascular risk factors [9-13]. Additionally, there are about 838,000 illiterate adults living in Portugal (9% of the population, mostly among the elderly and rural populations) based on the 2001 Census, with illiteracy-dependent limitation of the cognitive reserve being a proven risk factor for the earlier onset of dementia [14]. Portugal is hence supposed to have social, cultural and economical idiosyncrasies which could hypothetically justify a high prevalence of cognitive impairment and dementia, although no population study has ever been taken to prove so. Also, significant geographic disparities are acknowledged to occur in Portugal, with a meaningful part of the population residing in rural, culturally and economically disfavoured areas. Recognition of the effects of such contrasts in the risk of cognitive impairment may justify different interventions and allocation of resources in rural and urban areas.

In this context, a population-based longitudinal study (POLSCI) was set up in Northern Portugal to estimate the prevalence and incidence of dementia and CIND in rural and urban populations aged 55-79 years. The population is younger than usually targeted in most prevalence studies since one of the obiectives of the follow-up study is to elucidate about early factors affecting detection/recognition of dementia in order to develop health prevention programs to minimize and delay the impact of the disease, as it has been remarked in other studies[15]. This article presents the results of the prevalence survey undertaken in 2003.

## Methods

**Figure 1:**
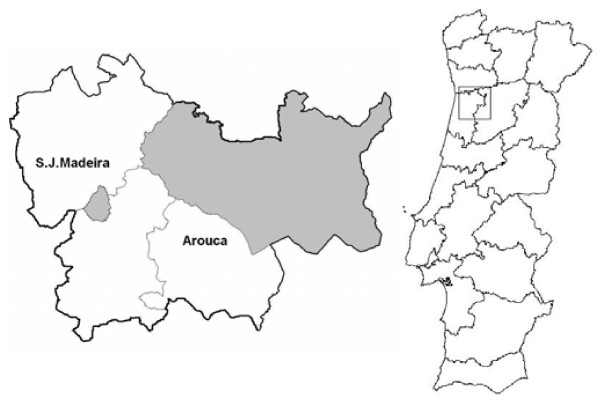
Map of Portugal showing the district and urban/rural areas (shaded) included in the study

The study was planned to involve representative rural and urban populations aged 55-79 years residing in Northern Portugal. The territorial unit "Entre Douro e Vouga" was chosen and within the area the two municipalities representing in the 2001 Census socio-demographic extremes in terms of population density (73 vs. 2.7161 inhabitants/km^2^), number of administrative divisions (20 vs. 1), main occupations (6.1% vs. 0.2% in farming and 50.5% vs. 69.6% in tertiary activities) and illiteracy (11.7% vs. 4.8%) - Arouca (rural) with 4,941 inhabitants and Sao João da Madeira (urban) with 4,117 inhabitants within the age range (Figure 1).

The minimum sample size was estimated for a confidence level of 95%, a level of precision of 2.5% and a "predicted" cognitive impairment prevalence of 16% in the rural community, since it was expected a higher prevalence than the reported in the Canadian Study of Health and Aging[3] in the 65-74 age-group (13.4%), similar to that reported for CIND for those 65 years and older. Reporting to data from the recent 2001 Census and keeping the urban-rural balance within the country (38.4% residing in cities and 61.6% outside), the required minimum sample size in the rural community was n = 710 (15%) and approximately n = 450 (11%) in the urban community (allowing for an identical precision of 2.5% if the estimated prevalence is 9%). Since the National Health Service has universal coverage, the two samples were drawn at random by computer from the population registered at the two community health centres by the end of 2002. Moreover, based on a preliminary study a relatively low response rate (50-60%) was to be expected [16] and so reserve samples were created from which replacements were selected in case of non-response. The study was planned to be completed in two consecutive three-month periods, starting in July 2003 in Arouca and ending by December 2003 in Sao João da Madeira. All persons examined as part of the study were informed of the scope of the project and gave an informed consent. All study procedures and ethical aspects were approved by the Hospital S. Sebastião ethical committee and the Health Centre directors.

### First Phase - Screening interview

Sampled subjects were sent written letters describing the survey objective and welcoming their participation. They were further contacted by phone to announce the screening interview at the community health centre and were asked to come with a close friend or family member. The interview lasted 45-60 minutes and was conducted by the same team of trained psychologists in the rural and urban areas. Besides socio-demographic information, questions about the current health status and medication were included, namely information about vascular risk factors and neurological/psychiatric diseases. The short version of the Geriatric depression scale [17] and the CAGE questionnaire [18] were used to screen depression and alcoholic habits. As part of the protocol designed, Subjective memory complaints (SMC) [19] were listed and the Blessed Dementia Scale (BDS)[20] and the Mini-Mental State Examination (MMSE)[21] were applied. The Blessed Dementia Scale (BDS, Blessed G, 1968) based on a close informant assesses functional difficulties in several daily activities, change in habits (eating, dressing and sphincter control) and mood (personality, behaviour, interests and emotions) with scores within a 0-28 scale. The cut-off of 4 has high validity (sensitivity = 95% and specificity = 84%) and the overall score correlates with the patient's neuropsychological performance [22].The neuropsychological evaluation comprised a set of tests from the "Lisbon Battery" formally translated, adapted and validated for the Portuguese population: the digit span for attention and working memory, logical memory I and II (spontaneous and delayed), paired-associate learning, visual memory, verbal memory with interference, categorical verbal fluency (food products) and naming [23].

### Second Phase - Neurological consultation

Screening criteria for dementia were set for an abnormal MMSE score or a BDS score of 4 points or higher. Cut-off points for the MMSE were 15 for illiterate persons, 22 for 1-11 years of education and 27 for 12 years or more, based on the validated Portuguese adaptation [24]. To establish screening criteria for CIND, MMSE scores were analysed in the rural and urban samples separately according to age and educational level, after excluding participants screening positive for dementia. This analysis was conducted in a sample of 1049 participants, (643 rural and 406 urban) and since age was not a significant source of variation after controlling for education, the cut-off points were set at 1 standard deviation bellow the sample's mean according to education level - 18 for illiterate, 24 for 1-3 years, 26 for 4 years and 27 for 12 or more years - and were identical for rural and urban samples.

All participants with suspected cognitive impairment (dementia or CIND) after the initial screen or whose information was not enough for applying the previous criteria were called to attend the second phase clinical examination at the Hospital S. Sebastião, including CT scanning and laboratory analysis whenever necessary. The health centre and hospital records were reviewed to check inconsistency on current health status/medication. The definite diagnosis for those screening positive in the first phase was established by consensus between the neurologists and the psychological coordinator.

### Criteria for CIND, dementia and subtypes

Dementia was diagnosed according to the DSM-IV-TR [1]. Following the definitions used in the Canadian Study oh Health and Aging [2,3], diagnoses of CIND were based on exclusion of dementia and the presence of various categories of impairment identified either in the clinical examination or in the neuropsychological tests. The categories considered were: general vascular, depression, cerebrovascular, alcohol or drug abuse, traumatic brain injury, sociocultural, psychiatric conditions, mental retardation and other neurological conditions. Whenever no medical, neurological/psychiatric condition was found for explaining cognitive impairment, it was considered cognitive impairment with no other condition (NOC).

### Data analysis

In order to analyse possible non-participation bias, the distribution by gender and age of participants and non-participants in both communities was compared using the chi-square test. The prevalence estimates of cognitive impairment according to diagnosis, socio-demographic characteristics and comorbidity, namely number of vascular risk factors, depression, cerebrovascular disease and other disease in urban and rural populations are presented and the corresponding 95% confidence limits (CI) were calculated by the Wilson "score" method [25]. The rural/urban prevalence ratios (PR) of cognitive impairment were used to compare the prevalence in both settings. Since sample weights represent the rural/urban balance in the overall Portuguese population, the overall prevalence estimates of dementia and CIND were calculated from the respondents' sample. Logistic regression models were used to adjust for the presence of multiple factors to ascertain the independent factors associated with dementia and CIND. Besides the main effects models, the importance of rural/urban environment was tested by including all interaction terms involving residence and the remaining variables in the model using a stepwise procedure. A probability value of 0.05 was used as the limit for Type I error (wrongly rejecting the null hypothesis).

## Results

**Figure 2:**
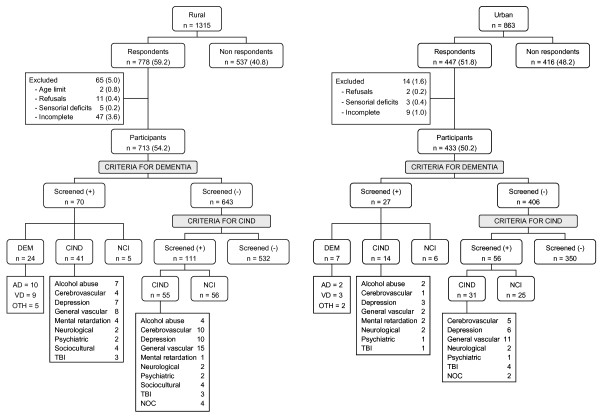
**Sample, participants and cognitive impairment classification.** General vascular: with at least one vascular risk factor (high blood pressure, cardiac disease, diabetes and dyslipidemia); TBI: traumatic brain injury; NOC: no other condition

**Figure 3:**
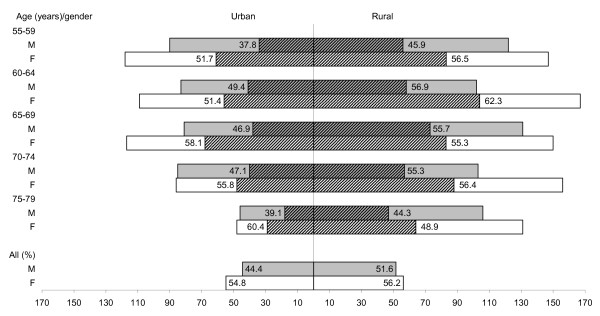
**Participants (n = 1146) by municipality, gender and age (%) (shaded areas)**. Total bars correspond to sample size in strata.

After a six-month period, 1315 persons (26.8% of the population) were contacted in the rural area (from July to December 2003), and 863 persons (21.0%) were contacted in the urban area (from March to May 2004) (Figure 2). The response rate was higher in the rural sample (59.2% vs. 51.8%; chi-square = 11.5, df = 1, p < 0.001), but since information was incomplete for 3.6% of rural respondents, the overall participation rate (52.6%) was not significantly different in the two communities (chi-square = 3.4, df = 1, p < 0.06). Though in the urban community women were more willing to participate than men (chi-square = 9.2, df = 1, p < 0.003), the distribution by age of male and female participants and non-participants was not significantly different both in the rural and urban communities (chi-square < 6.4, df = 4, p < 0.1) (Figure 3).

Based on the screening criteria for dementia, 97 persons were selected for the second phase neurological consultation (Figure 2). Among these, 31 were ultimately diagnosed with dementia and 55 with CIND. After applying criteria for CIND to the remaining 1049 participants, 167 screened positive and were selected for the second phase consultation - 86 of which were ultimately diagnosed as CIND. Overall, 172 persons were diagnosed as cognitively impaired, 16.8% in the rural community and 12.0% in the urban one, rural/urban PR = 1.40 (95% CI: 1.04-1.90) (Table 1). In general there is a higher prevalence of cognitive impairment in rural compared to urban populations for all population strata considered, reaching the highest prevalence ratios in the oldest (2.16), illiterate (2.05) and dependent (2.21). The prevalence ratios are not so high in the presence of comorbidity, ranging from 1.0 to 1.3; the only exception is in persons with two or more VRF, for which the prevalence is 24.8% in the rural area compared to 11.3% in the urban area. In these strata the prevalence in the rural community is remarkably high, almost one third of the population aged 75-79 years and just over one forth of illiterates or persons living alone compared with at most 15% of the urban population in the same strata. Another rural stratum particularly vulnerable are persons depending on others, near one half are cognitively impaired compared to 22.2% in the urban population. On the other hand the urban population not illiterate but with low levels of education seem more affected than the illiterates and the reverse trend happens in the rural population.

**Table 1:** Prevalence of cognitive impairment in rural and urban populations by socio-demographic characteristics and comorbidity

Characteristics	Rural				Urban				PR	95% CI
				
	N	No cases	Prev (%)	95% CI	N	No cases	Prev (%)	CI 95%		
All	713	120	16.8	14.3-19.8	433	52	12.0	9.3-15.4	1.40	1.04-1.90
Gender										
Men	291	43	14.8	11.2-19.3	171	16	9.4	5.8-14.7	1.58	0.92-2.72
Women	422	77	18.2	14.9-22.2	262	36	13.7	10.1-18.4	1.33	0.92-1.91
Age (years)										
55-64	294	30	10.2	7.2-14.2	192	18	9.4	6.0-14.3	1.09	0.63-1.90
65-74	301	52	17.3	13.4-22.0	194	27	13.9	9.8-19.5	1.24	0.81-1.91
75-79	118	38	32.2	24.5-41.1	47	7	14.9	7.4-27.7	2.16	1.04-4.50
Education										
Illiterate	189	50	26.5	20.7-33.2	31	4	12.9	5.1-28.9	2.05	0.80-5.28
1-3	226	39	17.3	12.9-22.7	103	21	20.4	13.7-29.2	0.85	0.53-1.36
4	273	28	10.3	7.2-14.4	206	20	9.7	6.4-14.5	1.06	0.61-1.82
5 or more	21	3	12.0	4.2-30.0	75	7	7.5	3.7-14.7	1.59	0.44-5.73
Residential status										
Living with the family	652	104	16.0	13.3-19.0	388	45	11.6	8.8-15.2	1.38	0.99-1.91
Living alone	57	15	26.3	16.7-38.9	42	6	14.3	6.7-27.8	1.84	0.78-4.35
Institutional residence	4	1	25.0	4.5-69.9	3	1	33.3	6.2-79.2	0.75	0.07-7.73
Occupation (present/late)										
Farming	402	76	18.9	15.4-23.0	19	6	31.6	15.4-54.0	0.60	0.30-1.20
Production	86	12	14.0	8.2-22.8	153	20	13.1	8.6-19.3	1.07	0.55-2.08
Personal care/serving	72	13	18.1	10.9-28.5	117	14	12.0	7.3-19.1	1.51	0.75-3.03
Sales/White collar	50	4	8.0	3.2-18.8	112	7	6.3	3.1-12.3	1.28	0.39-4.18
Housewife	103	15	14.6	9.0-22.6	32	5	15.6	6.9-31.8	0.93	0.37-2.37
Retired worker										
Yes	499	100	20.0	16.8-23.8	282	37	13.1	9.7-17.6	1.53	1.08-2.16
No	214	20	9.3	6.1-14.0	151	15	9.9	6.1-15.7	0.94	0.50-1.78
Depending on others										
Yes	53	26	49.1	36.1-62.1	18	4	22.2	9.0-45.2	2.21	0.89-5.47
No	660	94	14.2	11.8-17.1	415	48	11.6	8.8-15.0	1.23	0.89-1.71
Physical/Mental comorbidity										
Depression										
Yes	78	25	32.1	22.8-43.0	50	13	25.0	15.2-38.2	1.28	0.72-2.27
No	635	95	15.0	12.4-17.9	381	39	10.2	7.6-13.7	1.46	1.03-2.08
Cerebrovascular disease										
Yes	63	27	42.9	31.4-55.1	26	10	38.5	22.4-57.5	1.11	0.63-1.96
No	650	93	14.3	11.8-17.2	407	42	10.3	7.7-13.7	1.39	0.98-1.95
Other disease^†^										
Yes	124	47	37.9	29.8-46.7	59	21	35.6	24.6-48.3	1.07	0.71-1.61
No	589	73	12.4	10.0-15.3	374	31	8.3	5.9-11.5	1.50	1.00-2.23
No vascular risk factors^‡^										
0	383	50	13.1	10.0-16.8	237	19	8.0	5.2-12.2	1.63	0.99-2.69
1	229	45	19.7	15.0-25.3	134	26	19.4	13.6-26.9	1.01	0.66-1.56
2 or more	101	25	24.8	17.4-34.0	62	7	11.3	5.6-21.5	2.19	1.01-4.76

Prev: prevalence; PR: Rural/Urban prevalence ratio; ^†^at least one of: traumatic brain injury, alcohol abuse, psychiatric or other neurological disease; ^‡^Vascular risk factors include high blood pressure, cardiac disease, diabetes and dyslipidemia

### Prevalence and pattern of dementia and CIND

The overall prevalence of dementia was 2.7% (95% CI: 1.9-3.8), increasing steeply with age and decreasing with education level (Table 2). The prevalence of CIND was 12.3% (95% CI: 10.4-14.4) following dementia patterns in terms of age and education, but unlike dementia, tending to be higher in women compared to men, 14.2% vs. 9.5%. The prevalence of both CIND and dementia were higher in the rural area, in farmers, retired or dependent persons. Considering simultaneously the main effects of socio-demographic characteristics and comorbidity in a logistic regression model, the prevalence of dementia is associated with age, cerebrovascular disease and other neurological disease. The odds of dementia increases by 11% (95% CI: 3-18%) for an aging of one year and in persons with cerebrovascular disease (OR = 9.2, 95% CI: 4.1-20.5) or other diseases (OR = 3.6, 95% CI: 1.6-8.0) (Table 3). CIND was associated with the same factors but also with education, VFR and depression. Including in the model for CIND all interactions of factors with residential area in a stepwise procedure, there was a significant interaction of residence and age, that is, the rural/urban odds ratio of CIND increases by 1.07 (95% CI: 1.0-1.13) every year, corroborating the values shown in Table 1. Less educated persons (OR = 1.54, 95% CI: 1.02-2.33) and, as for dementia, persons with comorbid conditions are also at a higher risk of having CIND.

**Table 2:** Prevalence of CIND and dementia by socio-demographic characteristics and comorbidity

	CIND				Dementia		
		
Characteristics	N	No cases	Prev (%)	95% CI	No cases	Prev (%)	95% CI
All	1146	141	12.3	10.4-14.4	31	2.7	1.9-3.8
Residence							
Rural	713	96	13.5	11.1-16.2	24	3.4	2.3-5.0
Urban	433	45	10.4	7.9-13.6	7	1.6	0.8-3.3
Age (years)							
55-59	228	22	9.6	6.5-14.2	1	0.4	0.1-2.4
60-64	258	21	8.1	5.4-12.1	4	1.6	0.6-3.9
65-69	258	28	10.9	7.6-15.2	4	1.6	0.6-3.9
70-74	237	37	15.6	11.5-20.8	10	4.2	2.3-7.6
75-79	165	33	20.0	14.6-26.8	12	7.3	4.2-12.3
Gender							
Men	462	44	9.5	7.2-12.5	15	3.2	2.0-5.3
Women	684	97	14.2	11.8-17.0	16	2.3	1.5-3.8
Education (years)							
Illiterate	220	45	20.5	15.6-26.3	9	4.1	2.2-7.6
1-3	329	47	14.3	10.9-18.5	13	4.0	2.3-6.6
4	479	41	8.5	6.3-11.4	7	1.5	0.7-3.0
5 or more	118	8	6.8	3.5-12.8	2	1.7	0.5-6.0
Residential status							
Living alone	99	20	20.2	13.5-29.2	1	1.0	0.2-5.5
Living with the family	1040	119	11.4	9.7-13.5	30	2.9	2.0-4.1
Institutional residence	7	2	28.6	8.2-64.1	0	0.0	0.0-35.4
Occupation (present/late)							
Farming	421	64	15.2	12.1-18.9	18	4.3	2.7-6.7
Production	239	27	11.3	7.9-15.9	5	2.1	0.9-4.8
Personal care & serving	189	24	12.7	8.7-18.2	3	1.6	0.5-4.6
Sales/White collar	162	9	5.6	3.0-10.2	2	1.2	0.3-4.4
Housewife (not rewarded)	135	17	12.6	8.0-19.2	3	2.2	0.8-6.3
Retired worker							
Yes	781	110	14.1	11.8-16.7	27	3.5	2.4-5.0
No	365	31	8.5	6.1-11.8	4	1.1	0.4-2.8
Depending on others							
Yes	71	20	28.2	19.0-39.5	10	14.1	7.8-24.0
No	1075	121	11.3	9.5-13.3	21	2.0	1.3-3.0
Physical/Mental comorbidity							
Depression							
Yes	130	34	26.2	19.4-34.3	4	3.1	1.2-7.7
No	1016	107	10.5	8.8-12.6	27	2.7	1.8-3.8
Cerebrovascular disease							
Yes	89	22	24.7	16.9-34.6	15	16.9	10.5-26.0
No	1057	119	11.3	9.5-13.3	16	1.5	0.9-2.4
Other disease^†^							
Yes	183	55	30.1	23.9-37.1	13	7.1	4.2-11.8
No	963	86	8.9	7.3-10.9	18	1.9	1.2-2.9
No vascular risk factors^‡^							
0	620	57	9.2	7.2-11.7	12	1.9	1.1-3.4
1	363	57	15.7	12.3-19.8	14	3.9	2.3-6.4
2 or more	163	27	16.6	11.6-23.0	5	3.1	1.3-7.0

Prev: prevalence; ^†^at least one of: traumatic brain injury, alcohol abuse, psychiatric or other neurological disease; ^‡^Vascular risk factors include high blood pressure, cardiac disease, diabetes and dyslipidemia

**Table 3:** Socio-demographic characteristics and comorbidity associated with CIND and dementia

Characteristics	CIND				Dementia	
		
	Main effects model		Interaction model		Main effects model	
	
	OR	95% CI	OR	95% CI	OR	95% CI
Age (years)	1.06^†^	1.03-1.09	1.01	0.96-1.07	1.11^†^	1.03-1.18
Residence						
Rural vs. Urban	1.20	0.80-1.79	0.02*	0.01-0.96	1.44	0.57-3.66
Gender						
Women vs. Men	1.42	0.94-2.13	1.41	0.94-2.13	0.80	0.35-1.81
Education (years)						
0-3 vs. 4 or more	1.54*	1.02-2.33	1.54*	1.02-2.33	1.42	0.56-3.59
VRF						
Yes vs. No	1.53*	1.05-2.22	1.53*	1.05-2.22	1.11	0.49-2.49
CVD						
Yes vs. No	4.15^‡^	2.48-6.96	4.13^‡^	2.46-6.93	9.23^‡^	4.16-20.5
Depression						
Yes vs. No	3.13^‡^	1.95-5.03	3.16^‡^	1.96-5.08	1.30	0.40-4.23
Other disease						
Yes vs. No	5.95^‡^	3.96-8.94	6.05^‡^	4.02-9.10	3.59^†^	1.61-8.00
Residence * Age			1.07*	1.00-1.13		

OR: odds ratio; VRF: vascular risk factors (include at least one of high blood pressure, cardiac disease, diabetes and dyslipidemia); CVD: cerebrovascular disease; Other disease: include at least one of traumatic brain injury, alcohol abuse, psychiatric or other neurological disease)

* p < 0.05; ^† ^p < 0.01; ^‡ ^p < 0.001

Among the 31 cases of dementia identified, equal proportions of AD and VaD were diagnosed, a prevalence of 10.5 per 1000 population, followed by dementia induced by alcohol abuse, 1.7 per 1000 population, and in three cases clinical information did not warrant a definite diagnosis (Table 4). Both AD and VaD were more prevalent in rural settings (R:U = 3.0 and 1.8, respectively), but while AD was more prevalent among women (F:M = 2.0) and patients had a median age of 75 years, VaD was more prevalent in men (F:M = 0.3) and patients had a median age of 70.4 years.

**Table 4:** Distribution of cognitive status and subcategories of CIND and dementia

Cognitive status	N	%	R:U	F:M	Age (years)	Education (years)	MMSE score
NCI	974		0.94	0.96	66.2 (5)	4.0 (1)	28.0 (2)
CIND	141		1.3	1.5	69.9 (6)	3.0 (2)	22.0 (3)
General vascular	36	25.5	1.1	1.8	70.1 (7)	3.0 (2)	23.5 (3)
Depression	26	18.4	1.1	8.1	69.6 (4)	3.0 (2)	23.0 (2)
Cerebrovascular	20	14.2	1.4	1.3	69.7 (5)	3.0 (2)	22.5 (4)
Alcohol abuse	13	9.2	3.3	0.1	69.5 (5)	1.0 (2)	19.0 (3)
Traumatic brain injury	11	7.8	0.7	0.4	68.6 (7)	2.0 (2)	22.0 (3)
Sociocultural	8	5.7	8|0	8|0	75.2 (5)	0.5 (1)	18.0 (3)
Other neurological	8	5.7	0.6	1.1	73.4 (3)	4.0 (3)	26.0 (4)
Mental retardation	7	5.0	1.5	7|0	65.4 (8)	0.0 (0)	14.0 (3)
Psychiatric	6	4.3	1.2	0.7	67.0 (7)	4.0 (3)	26.0 (3)
No other condition	6	4.3	1.2	3.4	66.9 (8)	2.5 (2)	23.5 (4)
Dementia	31		2.1	0.7	73.9 (4)	2.0 (2)	17.0 (4)
AD	12	38.7	3.0	2.0	75.0 (7)	2.0 (2)	18.5 (5)
Vascular	12	38.7	1.8	0.3	70.4 (4)	2.5 (2)	17.0 (4)
Alcohol	2	6.5	0.6	0|2	75.2 (4)	0.5 (1)	18.0 (3)
Parkinson	1	3.2	1|0	1|0	74.1	3.0	20.0
Rapidly progressive	1	3.2	1|0	0|1	71.5	11.0	24.0
Unclassifiable	3	12.9	3|0	1.4	76.7 (2)	1.0 (2)	12.0 (6)

R:U - rural urban prevalence ratio; F:M - female/male prevalence ratio; median and interquartile deviation for other variables; whenever category is empty the ratio is indicated by a vertical line (|); MMSE: Mini Mental State Examination

The more frequent categories of CIND were cumulative vascular risk factors (eg, hypertension, cardiac disease, diabetes and/or dyslipidemia) (31.4%), followed by depression (18.4%) and cerebrovascular disease (more commonly previous stroke) (14.2%). Socio-cultural isolation (5.7%) and other neurological/psychiatric diseases were less frequent. CIND categories had different patterns in terms of gender (chi-square = 38.2, df = 9, p < 0.007), education level (chi-square = 22.8, df = 9, p < 0.007) and MMSE score (chi-square > 37.1, df = 9, p < 0.001). While depression was more prevalent in women (F:M = 8.1) and sociocultural isolation or mental retardation were only observed in women, traumatic brain injury or alcohol abuse were more prevalent in men (F:M = 0.1 and 0.4, respectively), the later more prevalent in rural areas (R:U = 3.3). Education level and MMSE performance tend to correlate among CIND persons, outlining two relatively homogeneous groups: low MMSE scores and low education level in the socio-cultural, mental retardation and alcohol abuse categories, and relatively high MMSE scores and education level in persons with psychiatric/neurological diseases. The remaining categories attained medium values for both variables.

## Discussion

The adoption of preventive strategies and rational allocation of resources to lessen the impact of dementia in the community population depend mostly on knowing the prevalence of cognitive impairment and the relative importance of its determinants. Given the lack of epidemiological data in our country, the POLSCI study was set up to estimate the prevalence and incidence of cognitive impairment in rural and urban populations from Northern Portugal, with the present paper reporting on the results from the first stage prevalence survey undertaken in 2003. As expected the prevalence of cognitive impairment was higher in rural compared to urban populations, the prevalence more than doubling in those aged 75-79 years and in those with two or more vascular risk factors. This pattern is replicated in CIND and dementia. Age, education level, vascular risk factors and co-morbid conditions (cerebrovascular disease, depression and other neurological/psychiatric disease) were determining factors of CIND both in rural or urban populations. Nevertheless the contrast between rural and urban environments is evidenced throughout the age span, the estimated rural/urban relative risk of CIND increasing with age, that is, a similar risk in the urban compared with the rural youngest populations and a higher risk in the rural compared with the urban oldest populations. Independent predictors of dementia were age and associated co-morbidity, especially the 9 times higher risk of dementia among those with cerebrovascular disease. These results point out the relatively high importance of both vascular risk factors and cerebrovascular disease in the patterns of cognitive impairment in the studied population. Indeed AD and VaD are equally likely in this age band (55-79 years) and nearly 40% of all CIND persons have cumulative vascular risk factors or a past history of cerebrovascular disease, pointing not only to the fact that a vascular pathogenesis underlies most cognitive impairment, but also that vascular cognitive impairment is probably a continuum which can in many patients be manifested only as cognitive impairment no dementia. Indeed VaD and AD have been theorized to have a transition period between normal cognition and dementia, with preclinical impairment compromising performance in several cognitive tests without a formal functional handicap [26]. With stroke being the more frequent cause of death in Portugal, one has to admit that "vascular CIND" (vCIND) could also be relatively more prevalent in Portugal thus reinforcing the importance of a proactive prevention of modifiable vascular risk factors.

In accordance with previous studies the overall prevalence of both CIND and dementia increased with age and decreased in persons with higher levels of education [4,27-31]. The prevalence of dementia in the youngest is similar to that found in the Netherlands [32] and higher than in the USA[33] or UK[34] but among the eldest (75-79 years) is higher than the reported in most European countries [4,32,35-40]. The slightly higher prevalence of dementia in men (3.2 vs. 2.3%) may be linked to the AD and VaD balance, the former more common among men and the later among women [41]. This balance is rather unusual in other European countries (eg. AD:VaD ratios of 3 in Great Britain or 2 in Italy) and approaches the vascular-preponderant reports from Japan and China (AD:VaD = 0.6) [42]. Actually, ratios resembling the European trend wouldn't be possible even if one were to consider the 10% of demented patients with undetermined diagnosis as 'probable-AD'. In Japan, the higher prevalence of VaD has been attributed to the high salt intake, traditional Japanese diet, and high stroke mortality [43]. Similar features can be found in the Portuguese population, with a known higher salt intake and higher stroke incidences, as compared to other western European countries [6-8]. A high prevalence of vascular risk factors may be the underlying reason for this 'VaD boost', as 31.7% of the study's participants had one risk factor, and 14.2% had at least two. Vascular risk factors are, in fact, known to be highly prevalent in the Portuguese population, with more than half of the Portuguese aged 65-84 years having hypertension, 17.8% having diabetes, and more than 5% already having had a stroke or myocardial infarction[8]. As in other studies, either in Caucasian or Oriental populations [43,44], patients with VaD were younger than those with AD (median age: 70.4 vs 75.0 years).

Unlike dementia, CIND was more prevalent in women (14.2 vs. 9.5%) as in other studies [30,31]. Illiteracy was more frequent in women than men (23.1 vs 13.4%) and may be partially responsible for the female preponderance in CIND, since association with socio-cultural factors and mental retardation were specific of women. Anyway vascular risk factors were actually the most common aetiological association for CIND in both men and women, illustrating their widespread effects. Other "organic" causes of CIND (as alcoholism and TBI) were more common among men, and CIND with no associated "organic" co-morbidity (depression, sociocultural isolation) was more frequent among women. The latter follows the AD pattern, leading to the hypothesis of a more purely degenerative pathogenesis of the CIND-dementia continuum in many women. Interestingly, other authors have pointed out that the true importance of vCIND cases has often been underappreciated, since it has been demonstrated that it can associate with rates of institutionalization and mortality similar to the ones found in AD [45]. In fact, vCIND must be faced as the most prevalent form of vascular cognitive impairment, also comprising VaD and mixed AD-VaD.

Another interesting finding was that the prevalence of dementia more than doubled in population strata without formal education (4.1 vs 1.7%) and half of demented persons had just two years of formal education. Illiteracy-imposed restriction of the cognitive reserve is a proven cause for an earlier onset of dementia, with low-educated persons demonstrating less tolerance to the neuropathologic effects of dementia and presenting clinically sooner [14]. In fact, illiteracy and unskilled occupations have been widely described in the literature as risk factors for dementia and are known to be heavily present in our population, especially in rural areas [28,29]. According to the 2001 Census, 28.7% of the Portuguese population has at most 3 years of formal education, and as much as 19.2% of them are illiterate, these proportions increasing to 31.7% and 26.5%, respectively, in rural populations. It has also been argued that, because of a less intellectually-demanding context in low-income areas, functional impairment may go unnoticed for a longer time, erroneously increasing the prevalence of dementia in developed areas. Since functional impairment is taken as mandatory for the diagnosis of dementia, some believe that current dementia criteria may therefore under-estimate the impact of cognitive impairment and the actual prevalence of dementia in low and middle income countries. This may be the reason why the rural/urban environment modifies the effect of age on CIND, that is, the rural/urban relative risk increasing with age. If this perspective were true, the present findings could conceal an even more expressive impact of rurality, low-education and low-income in the Portuguese population, especially at rural areas [46,47].

The POLSCI study has nevertheless some limitations. Since the study primary goal was to assess the overall prevalence of cognitive impairment, CIND and dementia, sample size estimates were based on previous reports on the prevalence of cognitive impairment in Canadian populations aged 65-74 years[3]. Even admitting that sample sizes may appear relatively small comparing to previous studies (less than 1000 participants each), the authors acknowledge the lost of power in statistical tests and low precision of some estimates, but the limited resources allocated to the project constrained more ambitious goals. Even so to reach the required minimum sample size the study lasted for 9 months instead of the 6 months previously planned. Restriction to the 55-79 years age band also assumedly neglects all cases of cognitive impairment among the 'very old', justified not only by logistic constraints but more importantly by the predicted low response and participation rates, resulting from physical constraints and reduced collaboration on testing among the eldest. During the follow-up of these cohorts of rural and urban populations data on incidence will clarify the prevalence estimates obtained. The present results can only be assumed to represent the reality of a relatively young age-group still amenable of preventive measures, and therefore focused on cognitive impairment rather than dementia. Even so the participation rate (52.6%) was lower than expected and lower than in previous studies [16], which possibly results from targeting younger population strata than seen in most prevalence studies, with the youngest participants still employed and less predisposed for attending an appointment at the local health centre. In the urban community women were in general more willing to participate than men, and so if we assume that women are more prone to CIND or dementia than men, the urban overall prevalence might be overestimated. Other studies have nevertheless reported that before the age of 80 the incidence of dementia is higher in men[48], what might be a reason for an underestimation of the prevalence in the urban environment. Irrespective of the residential area it is more likely that this is the situation, since in general non-participants tend to be more cognitively impaired than participants [49]. Since age is the more important predictor of cognitive impairment and the age distribution of men and women participants and non-participants from both rural and urban areas was not significantly different and similar to the population of the 2001 Census, the overall estimates of dementia and CIND should mirror the urban/rural mix in whole Portugal.

## Conclusions

To our knowledge, this is the first study to assess the prevalence of cognitive loss in the Portuguese population, and the results corroborate the expected high prevalence of cognitive impairment (CIND and dementia), as much as the assumption that Portugal may indeed be a paradigmatic example for regional variations in dementia among other countries, both in terms of rural-urban environments and socio-cultural disparities. Besides the well recognized age and education effects, patterns of cognitive impairment are determined by the association between age and rurality as well as by vascular risk factors, with vascular dementia being more prevalent than in other European countries. On the whole cognitive impairment has the highest prevalence in rural elderly persons, with both vCIND and vascular dementia as predominant patterns. Inevitably the selection of contrasting socio-economical environments may generate a bias when generalizing for the whole Northern Portugal population disregarding the rural and urban counterparts, but is plainly justified as the project's aim was to uncover consequences of rural/urban disparities and socio-demographic inequities.

Further demonstration of variation in prevalence and incidence estimates and patterns of cognitive impairment according to educational/economical characteristics and vascular risk profile in Northern Portugal should be potentially helpful for estimating the "preventable fraction" of dementia in the community, since it is recognized that only a small fraction of CIND persons will progress to dementia over a short time span. Hopefully, the illiteracy gap between urban and rural populations will soon fade away, but vascular risk factors need special attention from the Public Health Authorities, by adopting proactive early preventing measures.

## Competing interests

The authors declare that they have no competing interests.

## Authors' contributions

BN participated in the study design, acquisition and interpretation of data, drafting and revising the manuscript, RDS in data acquisition, analysis and drafting the manuscript, VTC in acquisition of data and revising the manuscript, JMR in revising the manuscript, JP in study design and data acquisition and MCS in study design, data analysis and interpretation and revising the manuscript. All authors read and approved the final manuscript.

## Pre-publication history

The pre-publication history for this paper can be accessed here:

http://www.biomedcentral.com/1471-2377/10/42/prepub

## References

[B1] American Psychiatric Association Diagnostic and statistical manual of mental disorders. Washington, DC 1994 4

[B2] Ebly EM Hogan DB Parhad IM Cognitive impairment of the nondemented elderly Arch Neurol 1995 52 612 19 7763211 10.1001/archneur.1995.00540300086018

[B3] Graham JE Rockwood K Beattie BL Eastwood R Gauthier S Tuokko H Mc Dowell I Prevalence and severity of cognitive impairment with and without dementia in an elderly population Lancet 1997 349 1793 96 10.1016/S0140-6736(97)01007-6 9269213

[B4] Hofman A Rocca WA Brayne C Breteler MM Clarke M Cooper B Copeland JRM Dartigues JF Droux AS Hagnell O Heeren TJ Engedal K Jonker C Lindesay J Lobo A Mann AH Mölsä PK Morgan K O'Connor DW Sulkava R Kay DWK Amaducci L for the EURODEM prevalence research group The prevalence of dementia in Europe: a collaborative study of 1980-1990 findings Int J Epidemiol 1991 20 736 748 10.1093/ije/20.3.736 1955260

[B5] Garcia C Costa C Guerreiro M Leitão O de Mendonça A Umbelino J An estimate of the prevalence of dementia and Alzheimer's disease in Portugal Acta Med Port 1994 7 487 91 7992654

[B6] Correia M Silva MR Matos I Magalhães R Lopes JC Ferro JM Silva MC Prospective Community-Based Study of Stroke in Northern Portugal. Incidence and case fatality in rural and urban populations Stroke 2004 35 2048 2053 10.1161/01.STR.0000137606.34301.13 15256683

[B7] Correia M Silva MR Magalhães R Guimarães L Silva MC Transient Ischemic Attacks in rural and urban Northern Portugal. Incidence and short-term prognosis Stroke 2006 37 50 55 10.1161/01.STR.0000195209.26543.8f 16322498

[B8] Instituto Nacional de Saúde Dr. Ricardo Jorge/Instituto Nacional de Estatística Fourth National Health Inquiry. Lisboa, Portugal 2005

[B9] Viswanathan A Rocca W Tzourio C Vascular risk factors and dementia. How to move forward? Neurology 2009 72 368 374 10.1212/01.wnl.0000341271.90478.8e 19171835 PMC2677504

[B10] De Ronchi D Palmer K Pioggiosi P Atti AR Berardi D Ferrari B Dalmonte E Fratiglioni L The combined effect of age, education, and stroke on dementia and cognitive impairment in the elderly Dementia Geriatr Cogn Disord 2007 24 266 73 10.1159/000107102 17700023

[B11] Purandare N Burns A Daly KJ Hardicre J Morris J Macfarlane G McCollum C Cerebral emboli as a potential cause of Alzheimer's disease and vascular dementia: case-control study BMJ 2006 332 1119 1124 10.1136/bmj.38814.696493.AE 16648133 PMC1459546

[B12] Prins ND van Dijk EJ den Heijer T Vermeer SE Koudstaal PJ Oudkerk M Hofman A Breteler MM Cerebral white matter lesions and the risk of dementia Arch Neurol 2004 61 1531 1534 10.1001/archneur.61.10.1531 15477506

[B13] Vermeer SE Prins ND den Heijer T Hofman A Koudstaal PJ Breteler MM Silent brain infarcts and the risk of dementia and cognitive decline NEngl J Med 2003 348 1215 1222 10.1056/NEJMoa022066 12660385

[B14] Paradise M Cooper C Livingston G Systematic review of the effect of education on survival in Alzheimer's disease Int Psychogeriatr 2009 21 1 25 32 10.1017/S1041610208008053 19026089

[B15] Jorm AF Dear KB Burgess NM Projections of future numbers of dementia cases in Australia with and without prevention Aust N Z J Psychiatry 2005 39 959 963 16343295 10.1080/j.1440-1614.2005.01713.x

[B16] Nunes B Cruz VT Pais J Mateus A Silva R Silva MC Rastreio populacional de demência e defeito cognitivo ligeiro nos concelhos de Matosinhos e de Arouca - populações e métodos do estudo piloto Sinapse 2004 4 26 33

[B17] Galaria II Casten RJ Rovner BW Development of a shorter version of the geriatric depression scale for visually impaired older patients Int Psychogeriatr 2000 12 435 443 10.1017/S1041610200006554 11263710

[B18] Ewing JA Detecting alcoholism. The CAGE questionnaire JAMA 1984 252 1905 1907 10.1001/jama.252.14.1905 6471323

[B19] Schmand B Jonker C Hooijer C Lindeboom J Subjective memory complaints may announce dementia Neurology 1996 46 1 121 125 8559359 10.1212/wnl.46.1.121

[B20] Blessed G Tomlinson BE Roth M The association between quantitative measures of dementia and of senile change in the cerebral grey matter of elderly subjects Br J Psychiatry 1968 114 797 811 10.1192/bjp.114.512.797 5662937

[B21] Folstein MF Folstein SE McHugh PR "Mini-mental state". A practical method for grading the cognitive state of patients for the clinician J Psychiatr Res 1975 12 189 198 10.1016/0022-3956(75)90026-6 1202204

[B22] Erkinjuntti T Hokkanen L Sulkava R Palo J The blessed dementia scale as a screening test for dementia Int J Geriatr Psychiatr 1988 4 267 273 10.1002/gps.930030406

[B23] Reis A Guerreiro M Petersson KM A sociodemographic and neuropsychological characterization of an illiterate population Appl Neuropsychol 2003 10 191 204 10.1207/s15324826an1004_1 14690800

[B24] Guerreiro M Contributo da Neuropsicologia para o estudo das Demências Phd thesis. Universidade de Lisboa, Faculdade de Medicina 1998

[B25] Newcombe RG Two-sided confidence intervals for the single proportion: comparison of seven methods Stat Med 1998 17 857 872 10.1002/(SICI)1097-0258(19980430)17:8<857::AID-SIM777>3.0.CO;2-E 9595616

[B26] Bäckman L Memory and cognition in preclinical dementia: what we know and what we do not know Can J Psychiatry 2008 53 354 60 18616855 10.1177/070674370805300604

[B27] Fratiglioni L DeRonchi D Agüero-Torres H World-wide prevalence and incidence of dementia Drugs and Aging 1999 15 365 375 10.2165/00002512-199915050-00004 10600044

[B28] Fratiglioni L Grut M Forsell Y Viitanen M Grafström M Holmen K Ericsson K Bäckman L Ahlbom A Winblad B Prevalence of Alzheimer's disease and other dementias in an elderly urban population: relationship with age, sex, and education Neurology 1991 41 1886 1892 1745343 10.1212/wnl.41.12.1886

[B29] Prencipe M Casini AR Ferretti C Lattanzio MT Fiorelli M Culasso F Prevalence of dementia in an elderly rural population: effects of age, sex, and education J Neurol Neurosurg Psychiatry 1996 60 628 633 10.1136/jnnp.60.6.628 8648328 PMC1073945

[B30] Cristina S Nicolosi A Hauser WA Leite MLC Gerosa E Nappi G The prevalence of dementia and cognitive deficit in a rural population of 2442 residents in northern Italy. A door-to-door survey European J Neurology 2001 8 595 600 10.1046/j.1468-1331.2001.00301.x 11784344

[B31] Prencipe M Santini M Casini A Pezzella F Scaldaferri N Culasso F Prevalence of non-dementing cognitive disturbances and their association with vascular risk factors in an elderly population J Neurol 2003 250 907 912 10.1007/s00415-003-1094-0 12928907

[B32] Ott A Breteler M Claus J van der Cammen T Grobbee D Prevalence of Alzheimer's disease and vascular dementia: association with education. The Rotterdam study BMJ 1995 310 970 973 7728032 10.1136/bmj.310.6985.970 PMC2549358

[B33] Kokmen E Beard CM Offord KP Kurland LT Prevalence of medically diagnosed dementia in a defined United States population: Rochester, Minnesota, January 1, 1975 Neurology 1989 39 73 76 10.1212/wnl.39.6.773 2725870

[B34] Harvey RJ Skelton-Robinson M Rossor MN The prevalence and causes of dementia in people under the age of 65 years J Neurol Neurosurg Psychiatry 2003 74 1206 1209 10.1136/jnnp.74.9.1206 12933919 PMC1738690

[B35] Rocca WA Bonaiuto S Lippi A Luciani P Turtú F Cavarzeran F Amaducci L Prevalence of clinically diagnosed Alzheimer's disease and other dementing disorders: a door-to-door survey in Appignano, Macerata Province, Italy Neurology 1990 40 626 631 2320236 10.1212/wnl.40.4.626

[B36] Vilalta-Franch J López-Pousa S Llinàs-Reglà Prevalencia de demencias en una zona rural. Estudio de Girona Rev Neurol 2000 30 1026 1032 10904947

[B37] Di Carlo A Baldereschi M Amaducci L Maggi S Grigoletto F Scarlato G Inzitari D Cognitive impairment without dementia in older people: prevalence, vascular risk factors, impact on disability. The Italian Longitudinal Study on Aging JAGS 2000 48 775 782 10.1111/j.1532-5415.2000.tb04752.x 10894316

[B38] Rocca WA Bonaiuto S Lippi A Luciani P Turtú F Cavarzeran F Amaducci L Prevalence of clinically diagnosed Alzheimerï¿½s disease and other dementing disorders: a door-to-door survey in Appignano, Macerata Province, Italy Neurology 1990 40 626 631 2320236 10.1212/wnl.40.4.626

[B39] Rocca WA Hofman A Brayne C Breteler MMB Clarke M Copeland JR Dartigues JF Engedal K Hagnell O Heeren TJ Jonker C Lindesay J Lobo A Mann AH Mölsä PK Morgan K O'Connor DW Droux AS Sulkava R Kay DWK Amaducci L The EURODEM- Prevalence Research Group Frequency and distribution of Alzheimer's disease in Europe: a collaborative study of 1980-1990 prevalence findings Ann Neurol 1991 30 381 390 10.1002/ana.410300310 1952826

[B40] Rocca WA Hofman A Brayne C Breteler MMB Copeland JRM Dartigues J-F Engedal K Hagnell O Heereen TJ Jonker C Lindesay J Lobo A Mann AH Mölsä PK Morgan K O' Connor DW da Silva Droux A Sulkava R Kay DWK Amaducci L The EURODEM- Prevalence Research Group The prevalence of vascular dementia in Europe: facts and fragments from 1980-1990 studies Ann Neurol 1991 30 817 824 10.1002/ana.410300611 1838681

[B41] Lobo A Launer LJ Fratiglioni L Andersen K Di Carlo A Breteler MM Copeland JR Dartigues JF Jagger C Martinez-Lage J Soininen H Hofman A Prevalence of dementia and major subtypes in Europe: A collaborative study of population-based cohorts Neurology 2000 54 Suppl 5 S4 S9 10854354

[B42] Jorm AF Cross-national comparisons of the occurrence of Alzheimer's disease and vascular dementia Eur Arch Psychiatry Clin Neurosci 1991 240 218 222 10.1007/BF02189530 1828995

[B43] Ineichen B Senile dementia in Japan: prevalence and response Soc Sci Med 1996 42 169 72 10.1016/0277-9536(95)00083-6 8928026

[B44] Fish M Bayer A Gallacher JEJ Bell T Pickering J Pedro S Dunstan FD Ben-Shlomo Y Ebrahim S Prevalence and pattern of cognitive impairment in a community cohort of men in South Wales: metholology and findings from the Caerphilly Prospective Study Neuroepidemiology 2008 30 25 33 10.1159/000115439 18259098

[B45] Rockwood K Wentzel C Hachinski V Hogan DB MacKnight C McDowell I for the Vascular Cognitive Impairment Investigators of the Canadian Study of Health and Aging Prevalence and outcomes of vascular cognitive impairment Neurology 2000 54 447 51 10668712 10.1212/wnl.54.2.447

[B46] Ferri CP Prince M Brayne C Brodaty H Fratiglioni L Ganguli M Hall K Hasegawa K Hendrie H Huang Y Jorm A Mathers C Menezes PR Rimmer E Scazufca M for Alzheimer Disease International Global prevalence of dementia: a Delphi consensus study Lancet 2005 366 2122 2127 16360788 10.1016/S0140-6736(05)67889-0 PMC2850264

[B47] Kalaria RN Maestre GE Arizaga R Friedland RP Galasko D Hall K Luchsinger JA Ogunniyi A Perry EK Potocnik F Prince M Stewart R Wimo A Zhang Z Antuono P for the World Federation of Neurology Dementia Research Group Alzheimer's disease and vascular dementia in developing countries: prevalence, management, and risk factors Lancet Neurol 2008 7 812 826 10.1016/S1474-4422(08)70169-8 18667359 PMC2860610

[B48] Letenneur L Gilleron V Commenges D Helmer C Orgogozo JM Dartigues JF Are sex and educational level independent predictors od dementia and Alzheimer's diesease? Incidence data from the PAQUID project J Neurol Neurosurg Psychiatry 1999 66 177 183 10.1136/jnnp.66.2.177 10071096 PMC1736218

[B49] Matthews FE Chatfield M Freeman C McCracken C Brayne C MRC CFAS Attrition and bias in the MRC cognitive function and ageing study: an epidemiological investigation BMC Public Health 2004 4 10.1186/1471-2458-4-12 15113437 PMC419705

